# Does the New European Driving Cycle (NEDC) really fail to capture the NO_X_ emissions of diesel cars in Europe?^☆^

**DOI:** 10.1016/j.envpol.2016.12.050

**Published:** 2017-03

**Authors:** Bart Degraeuwe, Martin Weiss

**Affiliations:** a European Commission, Joint Research Centre, Directorate C – Energy, Transport and Climate, Air and Climate Unit, via E. Fermi 2749, 21027 Ispra, Italy; b European Commission, Joint Research Centre, Directorate C – Energy, Transport and Climate, Sustainable Transport Unit, via E. Fermi 2749, 21027 Ispra, Italy

**Keywords:** On-road NO_X_ emissions, Diesel cars, New European Driving Cycle (NEDC), Real-driving emissions, Defeat strategies

## Abstract

Tests with Portable Emissions Measurement Systems (PEMS) have demonstrated that diesel cars emit several times more NO_X_ on the road than during certification on the New European Driving Cycle (NEDC). Policy makers and scientists have attributed the discrepancy to the unrealistically low dynamics and the narrow temperature range of NEDC testing. Although widely accepted, this assumption was never been put under scientific scrutiny. Here, we demonstrate that the narrow NEDC test conditions explain only a small part of the elevated on-road NO_X_ emissions of diesel cars. For seven Euro 4–6 diesel cars, we filter from on-road driving those events that match the NEDC conditions in instantaneous speed, acceleration, CO_2_ emissions, and ambient temperature. The resulting on-road NO_X_ emissions exceed by 206% (median) those measured on the NEDC, whereas the NO_X_ emissions of all unfiltered on-road measurements exceed the NEDC emissions by 266% (median). Moreover, when applying the same filtering of on-road data to two other driving cycles (WLTP and CADC), the resulting on-road NO_X_ emissions exceed by only 13% (median) those measured over the respective cycles. This result demonstrates that our filtering method is accurate and robust. If neither the low dynamics nor the limited temperature range of NEDC testing can explain the elevated NO_X_ emissions of diesel cars, emissions control strategies used during NEDC testing must be inactive or modulated on the road, even if vehicles are driven under certification-like conditions. This points to defeat strategies that warrant further investigations by type-approval authorities and, in turn, limitations in the enforcement of the European vehicle emissions legislation by EU Member States. We suggest applying our method as a simple yet effective tool to screen and select vehicles for in-depth analysis by the competent certification authorities.

## Graphical abstract

**Figure:**
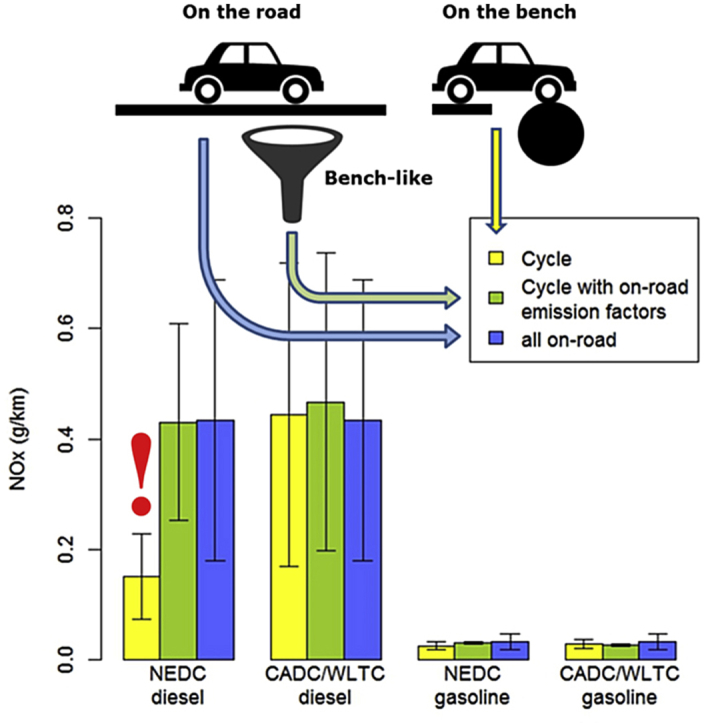

## Teaser

We demonstrate that diesel cars emit several times more NO_X_ on the road than on the NEDC certification cycle even when driven under the similar dynamic and temperature conditions.

## 1 Introduction

Investigations into the application of defeat devices and manipulated NO_X_ emissions have sparked a debate about the effectiveness of regulatory emissions testing. In view of persisting NO_2_ pollution in many cities across Europe (EEA, 2015), policy makers are more than ever urged to ensure that regulatory procedures (i) accurately capture the on-road emissions of vehicles and (ii) prevent the improper use of defeat strategies. Since 2007, on-road tests with Portable Emissions Measurement Systems (PEMS) in Europe have demonstrated that light-duty diesel vehicles, certified according to Euro 4 up to Euro 6 standards, emit a multiple of the amount of NO_X_ permitted by the respective emissions limit (Franco et al., 2014, Kadijk et al., 2015b, Ligterink et al., 2013, Rubino et al., 2007, Weiss et al., 2012). These exceedances were attributed to shortcomings in the type approval procedure, namely the low accelerations and the narrow ambient temperature range of 20–30 °C during vehicle certification with the New European Driving Cycle (NEDC) (ACEA, 2015, EEA, 2016, Weiss et al., 2012), and are currently addressed through the development of the Worldwide harmonized Light vehicles Test Procedure (WLTP) and the complementary Real-Driving Emissions (RDE) on-road test. With both procedures being at the verge of implementation, little attention has been paid to understand the actual origin of the elevated NO_X_ emissions of diesel cars on the road. We argue here that, contrary to the common view, insufficient driving dynamics and an overly narrow temperature range of NEDC testing may not be the root cause of the diesel-NO_X_ problem. Instead, we seek to demonstrate through a statistical analysis of emissions and driving data that large parts of the elevated on-road NO_X_ emissions can neither be explained by transient driving nor by the variability of ambient temperatures during on-road driving but may instead be related to the use of defeat strategies. We include in our analysis 10 light-duty vehicles that are tested on the NEDC in the laboratory and over various routes on the road. Our research can provide technical services and type-approval authorities with a simple yet effective tool for vehicle screening and may point to vehicles that warrant an in-depth assessment by the competent authorities.

## 2 Methods

### 2.1 Test vehicles and test routes

We test 10 light-duty vehicles of category M1 comprising 3 Euro 5 gasoline vehicles and 7 Euro 4–6 diesel vehicles (Table S1 in the Supplementary Information). With the exception of one, the Volkswagen Passat, all vehicles are obtained from third parties without involvement of the respective vehicle manufacturer. The emissions tests are conducted by the Vehicle Emissions Laboratories (VELA) of the Joint Research Centre (JRC) in Ispra, Italy. Market fuels, complying with Directive 2009/30/EC (EC, 2009) and the manufacturer's specifications for the operation of the respective vehicle, are used for all laboratory and on-road tests. In the laboratory, we conduct emission tests on a roller bench, manufactured by MAHA GmbH, with a 48 inch diameter, an inertia range of 454–4500 kg, and a maximum speed of 200 km/h. NO_X_ and CO_2_ emissions are sampled in Tedlar^®^ bags; component concentrations are determined with a Horiba MEXA-7400HTR-LE analyser in accordance with Regulation 83 (UNECE, 2015).

On the road, NO_X_ and CO_2_ emissions are measured with a Semtech^®^-DS or a Semtech^®^ Ecostar PEMS from Sensors Inc., comprising a tail-pipe attachment, heated exhaust line, exhaust flow meter, component analysers, a data logger to the vehicle network, and a GPS. The ambient temperature is measured at 1 Hz with a plug-and-play weather probe being part of the PEMS equipment (see also Weiss et al., 2011). In line with Regulation 2016/427 (EC, 2016), we calculate the instantaneous on-road NO_X_ and CO_2_ emissions [mg/s] at a frequency of 1 Hz by multiplying pollutant concentration, determined on a wet basis, and exhaust mass flow. The distance-specific emissions [mg/km] are then calculated by dividing the instantaneous emissions over the considered period by the distance driven in that period.

**Fig. 1:**
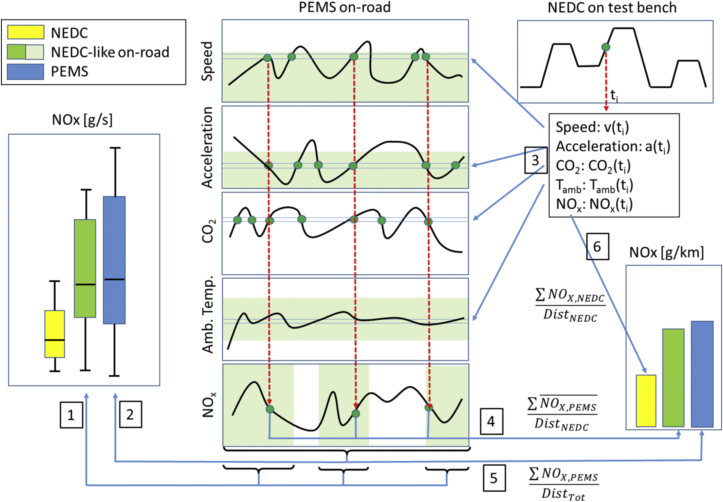
Stylized overview of the methodology. In the middle: the on-road data obtained with PEMS. On the left: box plots depicting the median and quartiles of the time-specific NO_X_ emission of (i) NEDC testing (yellow), (ii) NEDC-like on-road driving (green) and (iii) all on-road driving (blue). The whiskers represent 1.5 times the interquartile range. On the right: Distance-specific NO_X_ emissions over the NEDC based on actual test results (yellow), the application of on-road NO_X_ emission factors (green), and all on-road driving (blue). Each instantaneous speed-acceleration-CO_2_ combination occurring over the NEDC is matched with similar operating conditions on the road. The on-road NO_X_ emissions corresponding to each NEDC operating point are averaged (*NO_X,PEMS_*). The distance-specific NO_X_ emissions are finally determined by adding the averages of all operating points and dividing the outcome by the NEDC distance (*Dist_NEDC_*). (For interpretation of the references to colour in this figure legend, the reader is referred to the web version of this article.)

Our data analysis comprises two parts (Fig. 1). The first part serves the purpose of data exploration (Fig. 1, left). We make box plots of the instantaneous NO_X_ emissions [mg/s] of each vehicle during (i) NEDC testing in the laboratory (yellow box plot), (ii) selected on-road driving conditions that are similar to those of NEDC testing (green box plot, arrow 1), and (iii) on-road driving of all trips available over the various test routes (blue box plot, arrow 2). As conditions similar to NEDC testing we selected from all available on-road NO_X_ emissions [mg/s] those whose instantaneous (i) speed-acceleration combinations lay within the values attained by NEDC testing, (ii) ambient temperatures fall within the type-approval range of 20 to 30 °C and (iii) driving events occurred in flat terrain at road grades between −0.1 and + 0.1%. The results of this analysis are presented in box-plots depicting the median as well as the 2nd and 3rd quartile of the values. The box plots can already provide a first hint on the question whether deviations between laboratory and on-road NO_X_ emissions can be linked to variability in driving dynamics and ambient temperature.

The second part of our analysis consists of a second-by-second comparison of the NO_X_ emissions observed over the NEDC with those found on the road under similar operating conditions. This comparison requires controlling for ambient temperature and for the working points of the engine. Controlling for temperature is straightforward and achieved through excluding all on-road emissions data obtained outside of the type-approval temperature range of 20–30 °C. Controlling for the working points of the engine is more difficult and requires matching engine speed, angular acceleration and torque, as well as engine oil and coolant temperatures. Controlling for these parameters is standard practice in the bench testing of engines but it is less straight forward when testing passenger cars on the road as engine data are usually not available. We therefore approximate engine speed by vehicle speed. This approximation holds if vehicles are driven on the road and in the laboratory in the same gear at a given speed. Although this assumption may not always hold, averaging over a large amount of driving data likely renders deviations in the gear shift strategy as a random error. Second, we take vehicle acceleration as a proxy for the angular acceleration of the engine. Finally, we approximate torque by the CO_2_ emissions generated by the engine. At a given engine speed, torque is proportional to the instantaneous fuel consumption [g/s] and thus to CO_2_ emissions [g/s]. The chosen approximations make it possible to apply our method without the need to obtain engine data, e.g., through connection with the engine control unit, thereby decreasing the risk that vehicles detect an emissions test. Taken together, we characterize working points by vehicle speed, vehicle acceleration, and the instantaneous CO_2_ emissions - three parameters that are readily available from on-road emissions testing with PEMS.

We now take the second-by-second vehicle speed, acceleration, CO_2_ and NO_X_ emissions of each vehicle during NEDC testing in the laboratory and collect from our on-road PEMS data the NOx emissions for speed, acceleration, and CO_2_ events that match within a margin of 2 km/h, 0.02 m/s^2^ and 0.5 g CO_2_/km, respectively those found during NEDC testing (Fig. 1, arrow 3). In terms of ambient temperature, we make two choices. First, we apply our method only to on-road data recorded at the temperature interval permitted for type approval, i.e., 20 to 30 °C. This approach allows us to control for temperature as an explanatory variable for on-road NO_X_ emission levels but, at the same time, reduces the amount of test data available for our analysis. In fact, Table A1 in the Appendix shows that out of the seven diesel cars tested on the NEDC, five are driven on the road at type-approval temperatures. Two diesel cars, as well as the three gasoline cars, are tested on the road at ambient temperatures below the range of 20–30 °C. This in turn results in a lack or absence of emissions data in the range of permitted type-approval temperatures (see Table A1). Therefore, the analysis is also conducted without controlling for ambient temperature (see Table A1). After matching the working points, their average NO_X_ emissions are calculated (Fig. 1, arrow 4) and summed over an entire NEDC. This result is compared with the unfiltered PEMS data (Fig. 1, arrow 5) and the bench data (Fig. 1, arrow 6).

Given the availability of on average 23 ± 16 h of on-road driving data for each car comprising various trips and routes, we expect to find many speed-acceleration-CO_2_ points that correspond to those observed in the laboratory over the NEDC. In fact, driving conditions similar to those of the NEDC may not represent rare exceptions but rather common encounters on the road. Uphill and downhill driving is purposefully included in our data for the following reasons:

• For NEDC testing in the laboratory, a lower vehicle load than during on-road driving is used. This fact explains part of deviations in the fuel consumption and CO_2_ emissions determined during certification and found later by the consumers during normal vehicle use (Tietge et al., 2015, van Mensch et al., 2014). For this reason it is unlikely to find similar working points, characterized by speed, acceleration and CO_2_ emissions, in the laboratory and on a completely flat road. In fact, a car driven at constant speed at a given level of CO_2_ emissions over the NEDC would slow down on the road due to a higher real-world road load. As gravity helps to compensate the difference between road load in the laboratory and during actual real-world driving, the inclusion of slightly downward sloping roads can thus increase the likelihood of identifying on-road working points that match those in the laboratory.
• Related to the first point, the additional weight of PEMS equipment and a co-driver (around 100–150 kg) may be used as an argument to explain elevated NO_X_ emissions during PEMS tests. Using data from slight downhill driving makes it possible to correct for the effect of the weight added by the PEMS equipment to the car.

To verify the robustness of our method, we also apply it to the Common Artemis Driving Cycle (CADC) and the Worldwide harmonized Light-duty vehicles Test Cycle (WLTC) for selected vehicles. The CADC was developed to derive emission factors for air quality models and should yield levels of pollutant emissions similar to those found on the road (André, 2004). The WLTC will in the near future replace the NEDC for the certification of vehicles in Europe. All analyses are carried out with the open-source software package *R*. The script can be made available on request.

## 3 Results

**Fig. 2:**
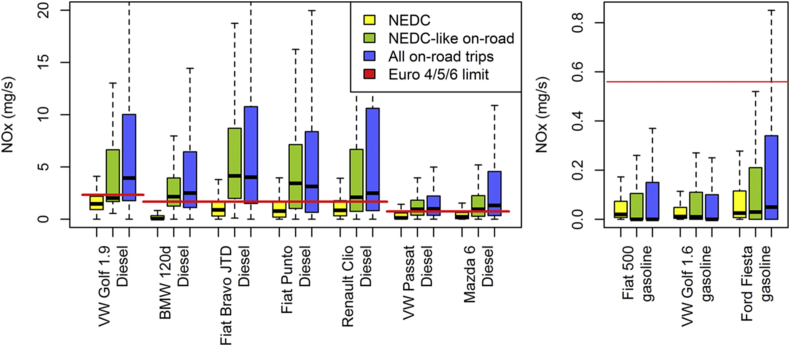
Box plots of time-specific NO_X_ emissions (mg/s) measured (i) over the NEDC (yellow), (ii) on the road under NEDC-like conditions characterized by vehicle speed, acceleration, road grade, and ambient temperature (green), and (iii) on the road as measured with PEMS over all available trips (blue); the red lines represent the applicable emission limits transformed into time-specific equivalents by multiplying the limit value with the distance of the NEDC (i.e., 11 km) and dividing the result by the duration of the NEDC (i.e., 1180 s). For the VW Golf, the Renault Clio, and the gasoline cars, no on-road measurements in the temperature range of 20–30 °C are available. (For interpretation of the references to colour in this figure legend, the reader is referred to the web version of this article.)

The tested diesel and gasoline cars tend to comply on the NEDC with their respective Euro 4–6 NO_X_ emission limits. In line with this observation, the bulk of the instantaneous NO_X_ emissions of all cars over the NEDC is located below the emissions limit (Fig. 2, yellow box plots). The picture differs for the on-road data; the bulk of the instantaneous NO_X_ emissions of diesel cars is located far above the limit (Fig. 2, blue box plots). Even when only NEDC-like conditions are selected, the box plots of on-road data for diesel cars show little overlap with those of the NEDC. If driving dynamics were responsible for the difference between NEDC and on-road NO_X_ emissions, one would expect that the green and yellow box plots span a similar range. For the gasoline cars, by contrast, the on-road NO_X_ emissions are higher than those over the NEDC but still lie below the emissions limit. If only NEDC-like driving conditions are selected, the box plots largely overlap with the ones of the NEDC (Fig. 2, green box plots). Although this part of the analysis does not yet account for the frequency of driving events, it already points to a strikingly different behaviour of diesel cars on the NEDC and during on-road driving under certification-like conditions.

**Fig. 3:**
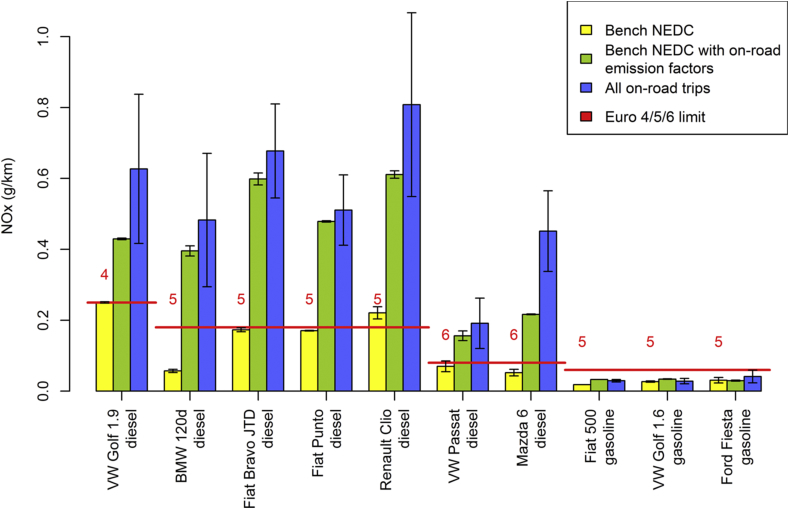
Distance-specific NO_X_ emissions determined (i) over the NEDC, (ii) over the NEDC applying on-road emission factors under NEDC-like conditions, and (iii) on the road over all available trips and routes; error bars represent the standard deviation of the distance-specific on-road NO_X_ emissions over various test routes. The red lines represent the applicable emission limits. (For interpretation of the references to colour in this figure legend, the reader is referred to the web version of this article.)

The second part of our analysis controls now in addition for engine operation and for the frequency of driving events (Fig. 3 and Table A1). When analysing how much NO_X_ a vehicle would emit over the NEDC if it showed on-road emission factors under NEDC-like driving conditions, we again observe striking differences between diesel and gasoline cars. For diesel cars, the calculated NO_X_ emissions exceed those measured on the NEDC with a median of 181% (5th %ile: 87%, 95th %ile: 315%). When controlling for ambient temperature, the results do not change. The median bias calculated on a sample of 5 out of 7 diesel cars is 206% (Table A1). When all PEMS data are considered, the diesel cars exceed their NEDC emissions by 266% (median, 5th %ile: 157%, 95th %ile: 744%; see Table A1). This is 20% more than for the NEDC-like PEMS data. If the differences between the NEDC and on-road emissions were explained by driving dynamics, one would expect the yellow and green bars in Fig. 3 to be of similar height. Instead, the green bars substantially exceed the yellow ones and lie in the range of the blue bars, depicting the average on-road NO_X_ emissions of diesel vehicles. For the gasoline vehicles, however, the calculated NO_X_ emissions exceed those measured on the NEDC only to a minor extent with a median value of only 26% (even if these vehicles are driven on the road at an ambient temperature below the certification range of 20–30 °C).

**Fig. 4:**
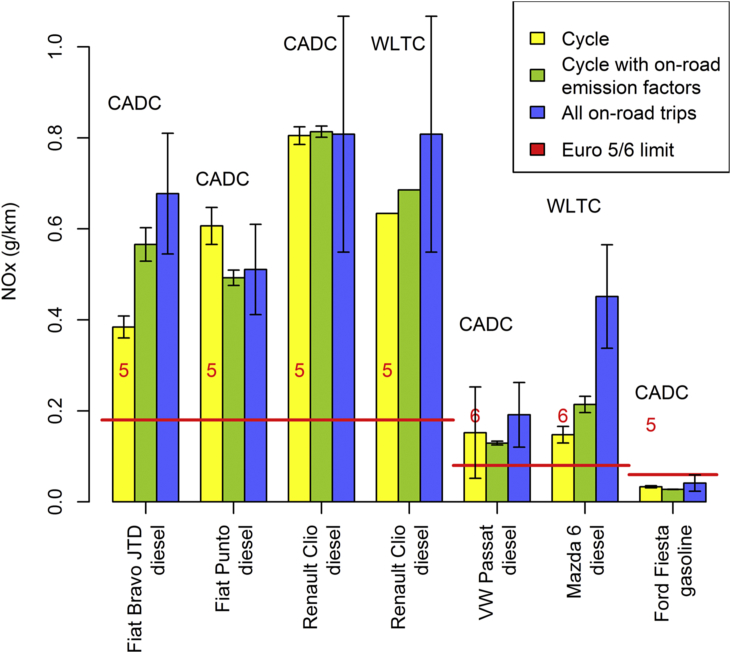
Distance-specific NO_X_ emissions determined (i) over the WLTC and CADC, (ii) over the WLTC and CADC applying on-road emission factors under cycle-like conditions, and (iii) on the road over all available trips and routes; error bars represent the standard deviation of the distance-specific on-road NO_X_ emissions over various test routes. The red lines represent the applicable emission limits. (For interpretation of the references to colour in this figure legend, the reader is referred to the web version of this article.)

Our findings for the NEDC also contrast with the observations for two alternative driving cycles, i.e., the CADC and the WLTC (Fig. 4 and Table A1). For diesel cars, the average NO_X_ emissions calculated with on-road emission factors exceed those measured in the laboratory by only 5% (median, 5th %ile: −18%, 95th %ile: 47%); when additionally controlling for ambient temperature, the bias remains similar (median: 13%, 5th %ile: −20%, 95th %ile: 42%). We regard these margins as indicative of the uncertainty of our calculation method.

## 4 Discussion

### 4.1 Uncertainties

We demonstrate that diesel cars show higher NO_X_ emissions on the road under NEDC-like conditions than during NEDC testing in the laboratory. Diesel cars appear to apply a different NO_X_ control strategy when tested on the NEDC compared to on-road driving even if dynamics and ambient temperature are controlled for. One could argue that the observed differences in the NO_X_ behaviour of diesel cars might result from measurement uncertainty or shortcomings in our calculation method. First, the PEMS and laboratory equipment used here comply with the requirements specified in Regulation 2016/427 (EC, 2016). Unpublished validation tests conducted by the Joint Research Centre with other vehicles suggest that the average deviation between PEMS and laboratory measurements is typically within 15% and thus negligible in view of findings presented here. Second, our calculations may be subject to uncertainties resulting from: (i) the assumption that the vehicle speed is a good proxy for engine speed, (ii) possible memory effects in the after-treatment system, (iii) not using coolant and oil temperature as selection criteria, (iv) not correcting for the time shift between engine out and tail pipe emissions, (v) not controlling for catalyst temperature or possible effects of road surface (vi) neglecting engine transients (e.g., due to gear shifting) that do not translate into vehicle accelerations. If these factors were important, one would expect to also find substantial emission differences when applying our method to driving cycles other than the NEDC. However, when doing so, we do not observe similarly large differences between the NO_X_ emissions of cycle-like on-road driving and those over the CADC and WLTC (see Table A1 in the Appendix). Additionally, we find that emission differences for gasoline cars are much smaller than those observed for diesel cars when comparing NEDC and NEDC-like on-road driving. This means the aforementioned uncertainties are small and that for diesel cars they cannot explain the observed differences in the NO_X_ emissions over the NEDC and NEDC-like on-road driving.

To substantiate this conclusion in detail, the sensitivity of our findings to the time alignment of signals was assessed. The alignment of emissions and driving events is typically subject to uncertainty as emissions reach the tail pipe with a time delay caused by the transportation of exhaust gases through the exhaust and after-treatment system. The emissions measured at the tailpipe hence correspond to an engine operating point that occurred typically 1–3 s before the actual emissions measurement, depending on engine load and exhaust mass flow (Ajtay and Weilenmann, 2004). To demonstrate the effect of time alignment, we look at those parts of the NEDC with a constant speed. The first and last 3 s of each constant speed part are not considered. For the 7 diesel cars the median bias remains large: 191% (5th %ile: 87%, 95th %ile: 298%) (Fig. S1 in the supplementary material). In an additional sensitivity analysis, we delayed the NO_X_ signal 1, 2 and 3 s. The results in Fig. S2 (see supplementary material) suggest that this has no significant effect on the results of our analysis. Hence, delay effects in the exhaust are negligible.

Moreover, our analysis does not differentiate between hot engine operation and cold-start, the latter being typically associated with elevated pollutant emissions as catalytic after-treatment systems operate at below-optimum temperature (Degraeuwe, 2007, Torregrosa et al., 2006). The cold-start effect is most pronounced for carbon monoxide and unburned hydrocarbons and less for NO_X_ that is predominantly formed at high combustion temperatures. Cold-start accounts for a larger portion of the NEDC than it does for on-road driving because a NEDC lasts only 20 min while a PEMS trip lasts around 2 h. We assess the impact of cold operation on our results, looking only at the extra-urban part of the NEDC. During this part, the coolant and oil temperature has stabilized. The results in Fig. S3 (see supplementary material) suggest that excluding cold-start, the median bias remains high: 210% (5th %ile: 81%, 95th %ile: 570%). Cold-start has thus a negligible effect, irrespectively whether one considers the vehicles equipped with exhaust gas recirculation only or the Volkswagen Passat equipped in addition with an SCR catalyst.

### 4.2 Reports by the type approval authorities (TAA)

As a consequence of the Volkswagen scandal, national type approval authorities were asked to investigate in depth the NO_X_ emissions of diesel cars. Recently the German, French, and British TAAs published reports with their findings (BMVI, 2016, Department for Transport, 2016, UTAC, 2016). The TAAs performed, among others, the following two tests: 1) the official NEDC on the test bench to check if cars were compliant with regulatory requirements and 2) an on-road test with a PEMS with a hot-start and the speed profile of the NEDC. Because the second test is similar to the filtering methodology presented in this paper, it is interesting to compare the results with our findings. In the British, French, and German reports, emissions were published for 56 Euro 5 and 78 Euro 6 cars. All cars, a few minor exceedances apart, passed the official NEDC test. However, the average NO_X_ emissions for the on-road NEDC were 809 mg/km (4.5 times the limit) for Euro 5 cars and 377 mg/km (4.7 times the limit) for Euro 6 cars. Sixteen per cent of the Euro 6 cars emitted over the on-road NEDC less than 80 mg NO_X_/km, which demonstrates that it is technically possible to comply with the Euro 6 NO_X_ emissions limit on the road. The TAAs contacted the manufactures for an explanation of the exceedances. The manufacturers argued that the high NO_X_ emissions during on-road NEDCs were due to the low ambient temperatures (average 10 °C, σ = 4 °C) that made it is necessary to reduce NO_X_ abatement strategies to protect the engine. We have to make four remarks on this argumentation: 1) The normal operating temperature of cars in Europe, taking into account daily activity patterns and population density, is 12 °C (σ = 4 °C; Malfettani et al., 2016). This means that the on-road NEDC tests were largely conducted under normal conditions of use in Europe in terms of ambient temperatures and at rather moderate driving dynamics. 2) The temperature argument put forward by the manufactures cannot be verified because no on-road NEDC tests were conducted at the type approval temperature in the 20–30 °C interval. This makes it impossible to assess if the elevated on-road NO_X_ emissions are in fact due to low ambient temperatures or rather due to the car driving on the road instead of the roller bench. 3) The conclusion that a low ambient temperature explains the high NO_X_ emissions of diesel cars contrasts with our findings. The cars for which PEMS data at the type approval temperature between 20 and 30 °C were available also showed high NO_X_ emissions at these temperatures (see Table A1). 4) When the NO_X_ emissions of the on-road NEDCs are plotted against the ambient temperatures there is a negative trend (see Fig. S4). For the Euro 6 cars, this trend is significant and average emissions converge towards the Euro 6 limit when ambient temperatures approach 20 °C. However, for Euro 5 cars, the trend is not significant and at 20 °C there is a considerable discontinuity between the NO_X_ emissions of on-road driving under NEDC-like conditions and the type approval with the NEDC of the same cars.

The German TAA (BMVI, 2016) also conducted real-driving emission (RDE) tests with PEMS. The comparison of the NO_X_ emission results from these tests with the results from the NEDC on-road tests supports our earlier conclusion according to which the NEDC captures, in principle, well the on-road NO_X_ emissions of diesel cars. A mixed-effects model explaining the NO_X_ emissions with ambient temperature and test type (i.e., on-road NEDC versus RDE test) was fitted to the data. For Euro 5 cars, there is no significant difference between on-road NEDC and RDE NO_X_ emissions. For Euro 6 cars, on-road NEDC emissions are 364 mg/km. The RDE emissions are 71 mg/km or 20% higher (95% CI: 4–140 mg/km). This result demonstrates that the difference in dynamicity between NEDC and real driving cannot fully explain elevated NO_X_ emissions under real driving.

### 4.3 Implications

Our analysis challenges the generally accepted view that the unrealistic driving profile and narrow temperature range of NEDC testing constitute the root cause of the diesel-NO_X_ problem in Europe. If the elevated NO_X_ emissions of diesel cars cannot be explained by the driving dynamics or the ambient temperature on the road, modifications of the NO_X_ control strategies during certification testing remain as likely explanation of our results. It appears that NO_X_ control is systematically decreased during on road driving, even if the diesel vehicles are driven under NEDC-like conditions. To identify how and based on which parameters the NO_X_ emissions control is modified is beyond the scope of this research. Various options exist, including decreased exhaust gas recirculation (EGR), modified fuel injection, decreased regeneration frequency in case the vehicle is equipped with a lean NO_X_ trap, or decrease urea dosing in case the vehicle is equipped with a selective catalytic reduction system. Recent investigations showed that on the road EGR rates and the efficiency of SCR systems tend to be considerably lower than on the NEDC in the laboratory (Kadijk et al., 2015a).

Our analysis is not intended to identify legally relevant cycle beating. Operating conditions (e.g., cold start at sub-zero temperatures) may necessitate reducing the performance of NO_X_ control technologies. Although Regulation 715/2007 (EC, 2007) prohibits defeat devices “that reduce the effectiveness of emission control systems”, exemptions are granted if (i) needed to protect the engine against damage or accidents and to ensure safe operation of the vehicle and if (ii) the defeat device does not function beyond the requirements of engine starting. In view of these provisions, our research suggests that the emissions testing with the NEDC is robust, assuming a rigorous enforcement of the regulatory provisions on defeat devices. Somewhat related, when driving four out of seven diesel cars under NEDC-like conditions on the road, the average NO_X_ emissions exceeded the on-board diagnostics (OBD) threshold beyond which elevated emissions should be signalled to the vehicle driver. The absence of such signalling at the observed NO_X_ levels may likewise warrant further attention.

Our method could be used by type approval authorities and independent organizations to screen for abnormal emission differences between NEDC and on-road driving. The small sample of vehicles covered here does not allow defining a precise threshold value beyond which the emissions behaviour of a car is suspicious. Yet, the average bias of our method of 2 ± 38% suggests that 80% could be used as first proxy of such a threshold.

To end this discussion, our findings need to be placed in the wider perspective of air quality and public health. The EU made a strong commitment to improve air quality by setting an annual average NO_2_ limit of 40 μg/m^3^ (EC, 2008). To reach this target, a NO_X_ emission limit for light-duty vehicles was first introduced in 1992 and made increasingly more stringent since. In practice, the NO_X_ emissions of diesel cars have not decreased accordingly, contributing a substantial share to the NO_2_ air pollution in most European cities (EEA, 2015). Several studies showed that reducing the diesel NO_X_ emissions can improve urban air quality (Degraeuwe et al., 2016, Kiesewetter et al., 2014). The technology to reduce NO_X_ emissions from diesel cars is available; the VW Passat in this study, some Euro 6 vehicles tested by Kadijk et al., 2015a, Kadijk et al., 2015b and 15% of the Euro 6 cars recently tested by national type approval authorities (BMVI, 2016, UTAC, 2016) demonstrate this. However, care has to be taken that the technology is used under all circumstances and that the resolution of the NO_X_ problem does not lead to other emission problems, such as ammonia slip following urea overdosing in SCR catalysts. In the meantime, cities will have to recur to other solutions like a ban of diesel cars in their centres and promoting public and active modes of transport.

## 5 Conclusions

From our research, we draw the following conclusions:

• The tested Euro 4–6 diesel cars exceed their respective NO_X_ emissions limit when driving on the road (median: +266%, 5th %ile: 157%, 95th %ile: 744%). This observation contrasts with the situation for gasoline cars whose on-road NO_X_ emissions remain below the applicable limit.
• Recalculating the NO_X_ emissions of diesel cars from NEDC testing with emission factors found under similar operating conditions on the road, results in a staggering median discrepancy of 206% (5th %ile: 114%, 95th %ile: 360%). The similar analysis for gasoline cars and for alternative driving cycles reveals good agreement between the NO_X_ emissions observed in the laboratory and under cycle-like conditions on the road. This observation suggests that our method is robust.
• A large part of the elevated on-road NO_X_ emissions of diesel cars can neither be explained by the insufficient dynamics of the NEDC nor by the narrow temperature range of type-approval testing. This result challenges the conventional wisdom according to which the deficient NEDC testing is at the root-cause of the diesel-NO_X_ problem, but instead points to the improper use of defeat strategies.
• The type approval authorities recently measured NO_X_ emissions during NEDC tests on the road. They found average exceedances of about 4.5 times the limit value at ambient temperatures that are normal for Europe.
• Our findings do not allow drawing conclusions regarding the legal validity of NO_X_ control strategies applied by the tested diesel cars. Yet, the observed NO_X_ emission patterns warrant further investigations by type-approval authorities. The research method presented here could be used for vehicle screening as a stepping stone to more tailored investigations by research institutions, technical services, and type approval authorities.
• Our findings suggest that emissions certification with the NEDC might be robust, provided regulatory provisions are rigorously enforced. This observation implies that the complementary Real-Driving Emissions (RDE) may likewise only be robust if backed by in-use conformity testing and comprehensive market surveillance.

## Figures and Tables

**Table A1:** NO_X_ emissions on the NEDC, CADC and WLTC, calculated emissions with on-road emission factors for the same cycles and emissions during on-road driving over all trips on various routes. The bias is calculated with the NO_X_ emissions on the cycle as reference as: NO_X_/NO_X__cycle–1. Comp stands for completeness, i.e. the percentage of operating points on the cycle for which similar on-road data are found on the road. After the median bias per vehicle group, the 5th and 95th percentile are given between brackets.

Car	Cycle	Number of tests	Fuel	Limit	NO_X_ limit	Laboratory	Cycle with on-road emission factors			Calculated with speed-acc-CO2-Temp			All on-road trips		
						NO_X_ (mg/km)	NO_X_ (mg/km)	Comp (%)	Bias (%)	NOx (mg/km)	Comp (%)	Bias (%)	NOx (mg/km)	Bias (%)	Temp (C)
VW Golf 1.9	NEDC	3	diesel	Euro4	250	250 ± 2	429 ± 2	98	72	–	25	–	627 ± 210	151	12 ± 3
BMW 120d	NEDC	3	diesel	Euro5	180	57 ± 5	396 ± 14	98	592	353 ± 16	95	517	483 ± 188	744	20 ± 3
Fiat Bravo JTD	NEDC	3	diesel	Euro5	180	173 ± 6	598 ± 17	98	246	572 ± 12	97	231	677 ± 133	291	25 ± 7
Fiat Punto	NEDC	3	diesel	Euro5	180	171 ± 1	479 ± 2	99	181	482 ± 4	96	182	511 ± 99	199	18 ± 2
Renault Clio	NEDC	7	diesel	Euro5	180	221 ± 17	611 ± 11	95	177	–	72	–	808 ± 259	266	17 ± 2
VW Passat	NEDC	5	diesel	Euro6	80	70 ± 15	156 ± 14	99	123	151 ± 13	98	115	191 ± 71	173	26 ± 4
Mazda 6	NEDC	2	diesel	Euro6	80	52 ± 9	217 ± 1	93	315	213 ± 1	93	308	451 ± 114	764	23 ± 4
							Median bias: 181 [87; 315]			Median bias: 206 [114; 360]			Median bias: 266 [157; 744]		
Fiat 500	NEDC	2	gasoline	Euro5	60	18 ± 0	33 ± 0	82	80	–	23	–	29 ± 4	62	10 ± 2
VW Golf 1.6	NEDC	2	gasoline	Euro5	60	27 ± 2	34 ± 1	98	26	–	28	–	28 ± 7	5	12 ± 2
Ford Fiesta	NEDC	3	gasoline	Euro5	60	31 ± 8	30 ± 1	98	−4	–	56	–	41 ± 18	34	13 ± 3
							Median bias: 26 [-1; 75]			Median bias:			Median bias: 34 [8; 59]		
Fiat Bravo JTD	CADC	6	diesel	Euro5	180	384 ± 24	566 ± 37	83	47	546 ± 36	82	42	677 ± 133	76	25 ± 7
Fiat Punto	CADC	5	diesel	Euro5	180	606 ± 41	493 ± 17	88	−19	481 ± 9	83	−21	511 ± 99	−16	18 ± 2
Renault Clio	CADC	8	diesel	Euro5	180	805 ± 19	813 ± 12	79	1	–	43	–	808 ± 259	0	17 ± 2
VW Passat	CADC	8	diesel	Euro6	80	152 ± 100	129 ± 4	92	−15	129 ± 4	88	−15	191 ± 71	26	26 ± 4
Renault Clio	WLTC	1	diesel	Euro5	180	634	685 ± 0	90	8	–	55	–	808 ± 259	27	17 ± 2
Mazda 6	WLTC	4	diesel	Euro6	80	148 ± 18	214 ± 18	90	45	209 ± 18	90	41	451 ± 114	206	23 ± 4
							Median bias: 5 [-18; 47]			Median bias: 13 [-20; 42]			Median bias: 27 [-12; 173]		
Ford Fiesta	CADC	4	gasoline	Euro5	60	33 ± 3	28 ± 0	89	−17	–	33	–	41 ± 18	24	13 ± 3
