# Supplementary material for: Does the New European Driving Cycle (NEDC) really fail to capture the NOX emissions of diesel cars in Europe?
Source: Environ Pollut. 2017 Mar;222:234–41. doi: 10.1016/j.envpol.2016.12.050 (PMC5304423; doi:10.1016/j.envpol.2016.12.050)
Supplement: Online data [file mmc1.docx]

# Supplementary material

Table S1: Specifications of test vehicles

| Vehicle | Category/Applicable emissions limit  (Description) | Model year | Odometer reading at test start [km] | Engine type/fuel  (After-treatment technology) | Engine capacity [cm^3^] (maximum engine power [kW]) | CO_2_ emissions at type approval [g/km] |
| --- | --- | --- | --- | --- | --- | --- |
| VW Golf 1.9 | M1/Euro 4  (compact passenger car) | 2004 | ~100,000 | Compression ignition/diesel  (EGR, OC) | 1896 (77) | 140 |
| BMW 120d | M1/Euro 5  (compact passenger car) | 2009 | 4,667 | Compression ignition/diesel  (EGR, OC, DPF) | 1995 (130) | 128 |
| Fiat Bravo JTD | M1/Euro 5  (compact passenger car) | 2009 | 3,408 | Compression ignition/diesel  (EGR, OC, DPF) | 1598 (88) | 129 |
| Fiat Punto | M1/Euro 5  (small passenger car) | 2010 | n.a. | Compression ignition/diesel  (EGR, OC, DPF) | 1248 (55) | 108 |
| Renault Clio | M1/Euro 5  (small passenger car) | 2012 | 1,600 | Compression ignition/diesel  (EGR, OC, DPF) | 1461 (55) | 106 |
| Volkswagen Passat | M1/Euro 6  (mid-size passenger car) | 2011 | ~22,000 | Compression ignition/diesel  (EGR, OC, DPF, SCR) | 1968 (103) | 155 |
| Mazda 6 | M1/Euro 6  (mid-size passenger car) | 2013 | n.a. | Compression ignition/diesel  (EGR, OC, DPF) | 2191 (129) | 129 |
| Fiat 500 | M1/Euro 5  (small passenger car) | 2009 | -10,000 | Spark ignition/gasoline  (TWC) | 1242 (51) | 119 |
| VW Golf 1.6 | M1/Euro 5  (compact passenger car) | 2009 | ~5,000 | Spark ignition/gasoline  (TWC) | 1595 (75) | 166 |
| Ford Fiesta | M1/Euro 5  (small passenger car) | 2010 | 1,909 | Spark ignition/gasoline  (TWC) | 1242 (44) | 127 |

n.a. – information not available; EGR – exhaust gas recirculation; OC – oxidation catalyst; DPF – diesel particulate filter; SCR – selective catalytic reduction catalyst; TWC – three-way catalyst

Table S2: Speed distribution, share of steep driving and duration of the on-road tests

|  | **Percentage of distance driven at speeds** | | | **Percentage of distance with**  **>5% up/down hill** | **Total distance (km)** | **Total Time (h)** |
| --- | --- | --- | --- | --- | --- | --- |
| **Vehicle** | **below 50 km/h** | **Between 50 and 90 km/h (%)** | **Above 90 km/h** |  |  |  |
| VW Golf 1.9 | 24 | 37 | 40 | 3 | 870 | 16 |
| BMW 120d | 25 | 30 | 45 | 5 | 1466 | 26 |
| Fiat Bravo JTD | 26 | 36 | 38 | 5 | 204 | 4 |
| Fiat Punto | 28 | 40 | 32 | 5 | 737 | 15 |
| Renault Clio | 24 | 31 | 45 | 6 | 1608 | 29 |
| VW Passat | 28 | 31 | 41 | 5 | 1386 | 26 |
| Mazda 6 | 32 | 38 | 29 | 7 | 800 | 17 |
| Fiat 500 | 37 | 38 | 25 | 8 | 2662 | 61 |
| VW Golf 1.6 | 36 | 43 | 22 | 7 | 1046 | 25 |
| Ford Fiesta | 33 | 30 | 37 | 10 | 476 | 10 |


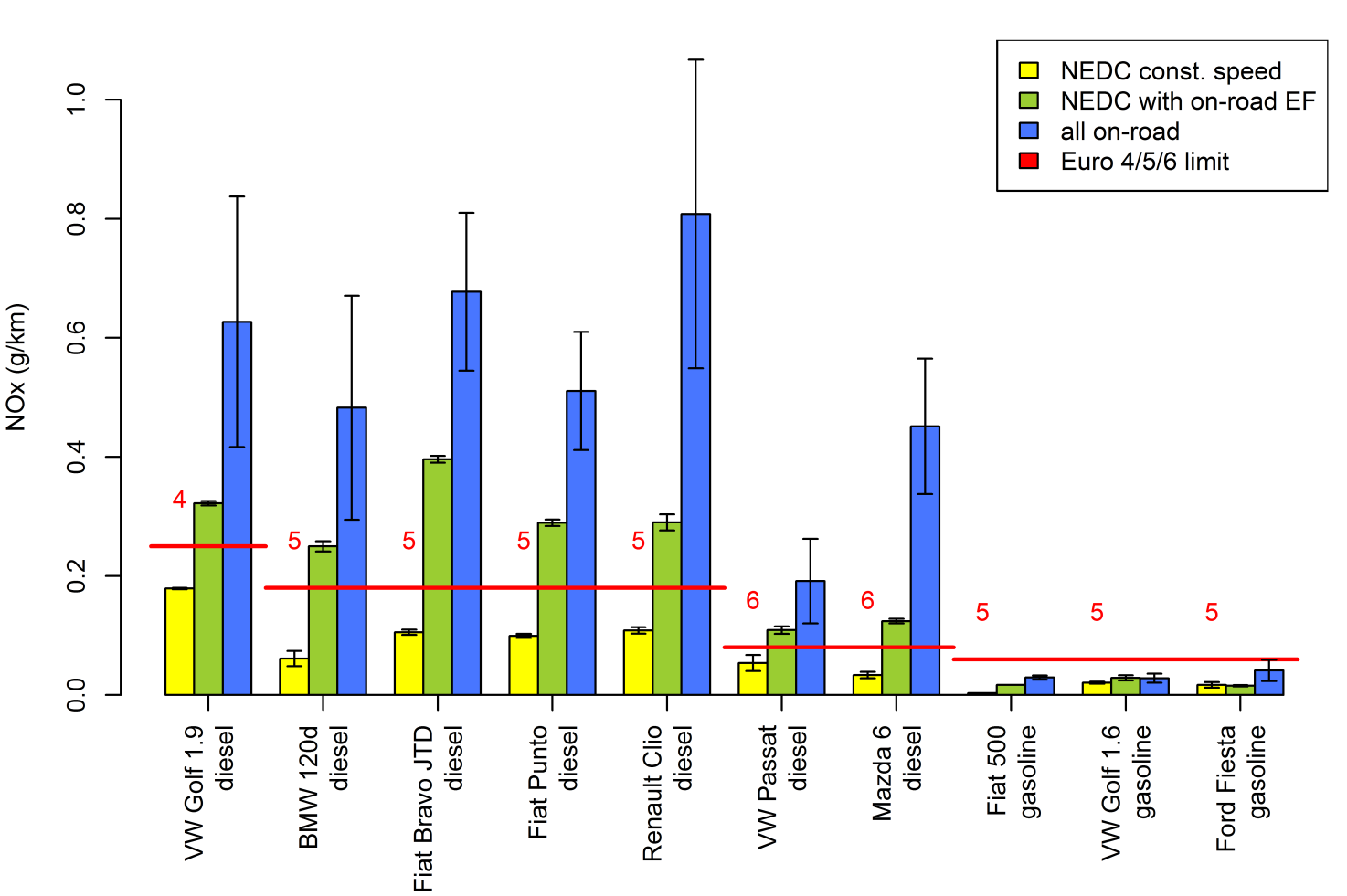


Figure S1: A comparison of NO_X_ emissions over constant speed parts of the NEDC and comparable on-road driving events; the results are similar to those found when comparing the NO_X_ emissions over the entire NEDC with those of NEDC-like on-road driving. Similarity is based on speed, acceleration and CO_2_ (ambient temperature is not considered here).


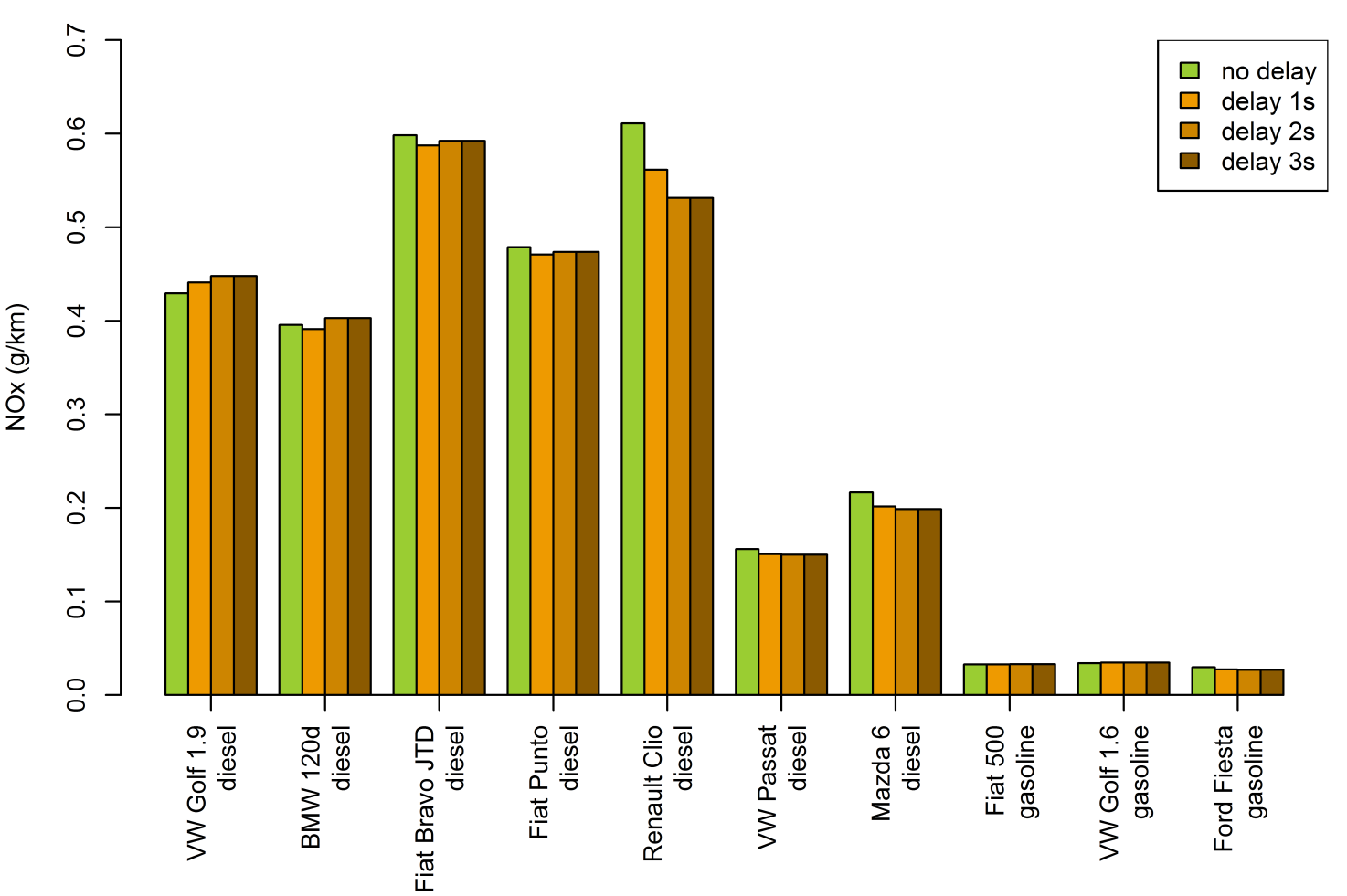


Figure S2: The effect of delay in the exhaust line on the calculated NO_X_ emissions over the NEDC with on-road emission factors. On-road emissions are selected based on instantaneous speed, acceleration and CO_2_ emissions. Delays of 0, 1, 2 and 3 seconds are applied to the NO_X_ concentration signal.


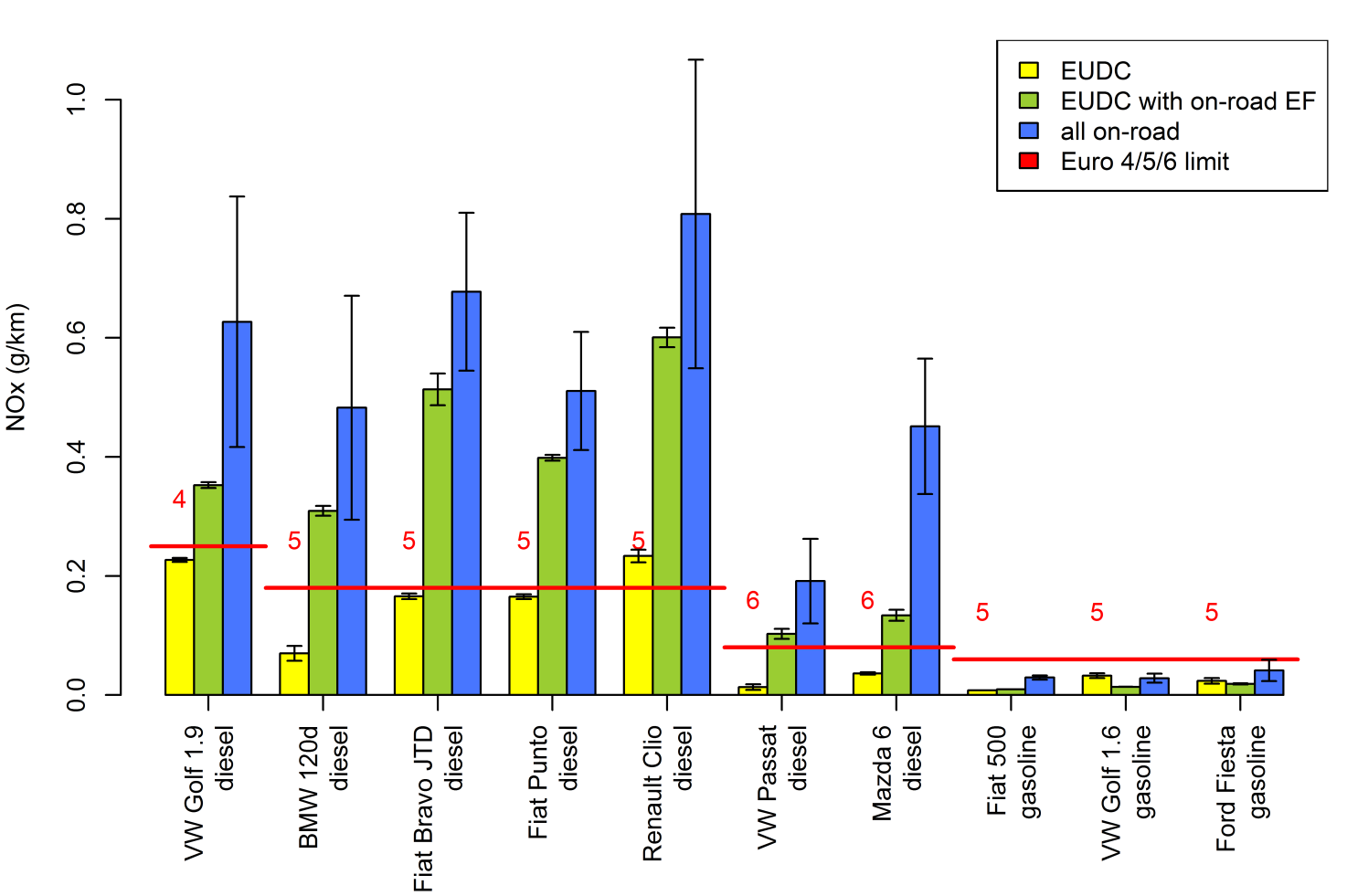


Figure S3: A comparison of NO_X_ emissions over the extra-urban part of the NEDC (EUDC) and comparable on-road driving events; the results are similar to those found when comparing the NO_X_ emissions over the entire NEDC with those of NEDC-like on-road driving. Similarity is based on speed, acceleration and CO_2_ emissions (ambient temperature is not considered here).


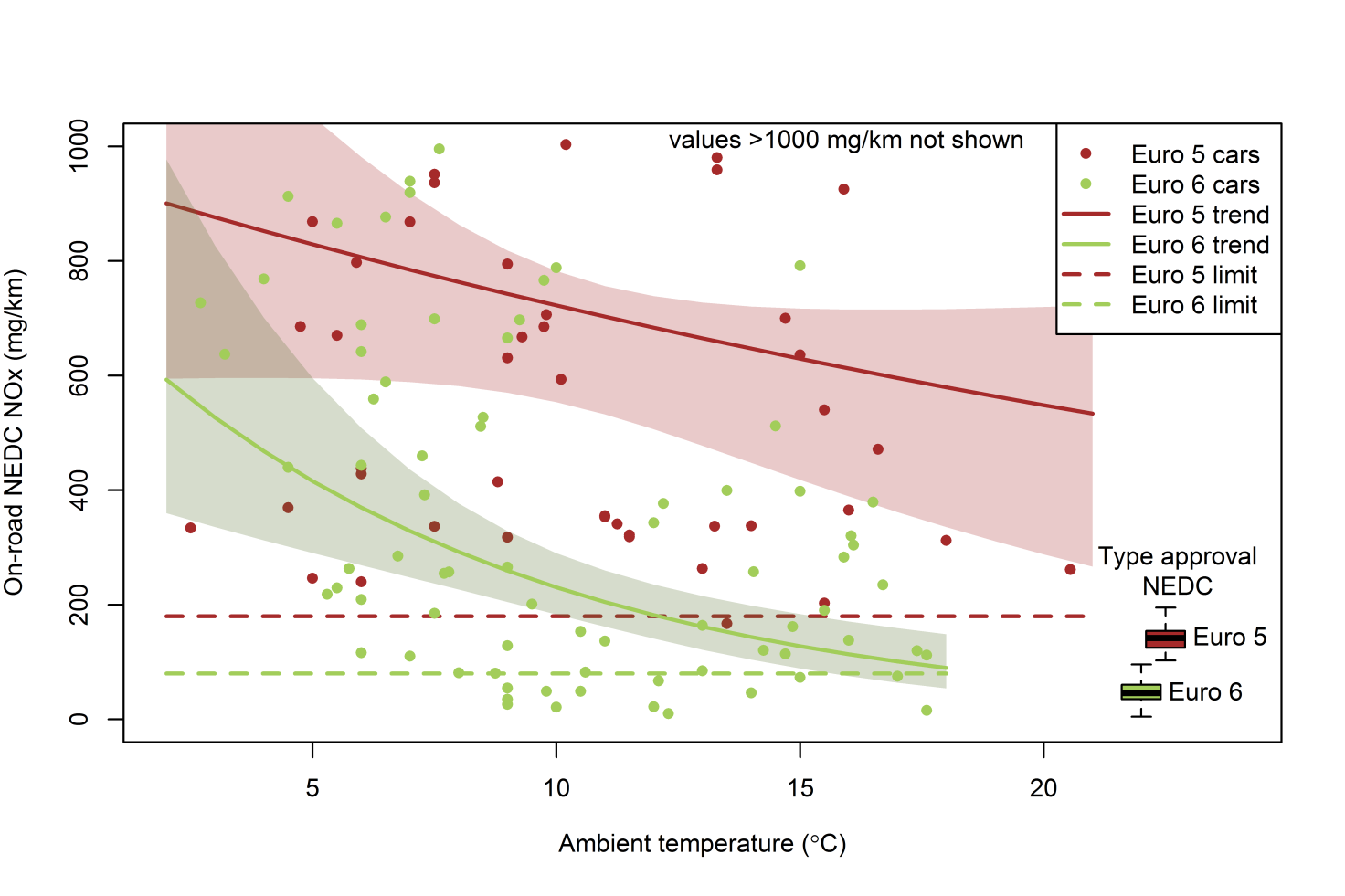


Figure S4: Temperature trend of NO_x_ emissions from on-road NEDC tests performed by British, German and French type approval authorities. The shaded areas are the confidence interval on the mean. The boxplots represent the type approval NO_x_ emissions of the same cars.
